# Novel Analysis on Aroma Compounds of Wine, Vinegar and Derived Products

**DOI:** 10.3390/foods10061245

**Published:** 2021-05-30

**Authors:** Enrique Durán-Guerrero, Remedios Castro

**Affiliations:** Analytical Chemistry Department, Faculty of Sciences-University Institute of Wine and Food Research (IVAGRO-CAIV), University of Cadiz, Agrifood Campus of International Excellence (CeiA3), Polígono Río San Pedro, s/n, 11510 Puerto Real, Spain; remedios.castro@uca.es

Aroma is one of the main responsible for the acceptance of oenological products such as wine, vinegar and derived products. Aroma compounds are produced during the winemaking process, and they can be affected by natural, geographical and human factors: raw material, alcoholic and acetic fermentation, aging, distillation, technological processes, etc. Therefore, it is very important the study and characterization of the aromatic fraction of these oenological beverages, in order to improve the quality of the final product.

Therefore, this special issue “*Novel Analysis on Aroma Compounds of Wine, Vinegar and Derived Products*” is focused on the recent research related to the study of the volatile composition of wine, vinegar and derived products from several different fields of science: oenology, chemistry, food science and technology, biochemistry, microbiology, biotechnology, chemical engineering, sensory analysis, etc. As a result, this special issue includes 12 valuable scientific contributions, 2 reviews and 10 original research works, which deal with the latest advances in both sensory and analytical tools to evaluate the effect of different techniques or winemaking stages on the oenological products’ aroma.

In this sense, Pérez-Jiménez et al. employed the in-mouth headspace sorptive extraction (HSSE) technique, which is based on the application of a polydimethylsiloxane (PDMS) coated bar in the mouth, after the sample intake, to perform the headspace intra-oral aroma extraction [1]. In this way, twenty-two wine aroma compounds were identified at three times: immediately after spitting out the sample, after 60 s, and finally, after 120 s. The different volatile compounds exhibited different release times, with low persistence for esters and linear alcohols. Muñoz-González et al. also used this methodology to evaluate the wine esters release and their perception under the influence of grape seed tannin extracts [2]. The authors concluded that the addition of this type of extract to wines could modify their aroma perception in a compound-dependent manner.

On the other hand, Tarasov et al. determined three sensory thresholds (detection, recognition, and rejection thresholds) for 1,1,6-Trimethyl-1,2-dihydronaphthalene (TDN) in Riesling wine using sensory analysis [3]. This volatile compound is related to kerosene aroma, and its recognition seems to be modulated by the wine serving temperature, with low temperatures facilitating it. Studying the winemaking of sweet wines by means of sensory analysis, Ruiz-Bejarano et al. established that the use of climate chambers in the elaboration of sweet wines seems to be an adequate alternative to the traditional method, allowing a total control of the process and producing very well-valued sweet wines [4].

From a sensory and instrumental point of view, Úbeda et al. studied the influence of bentonite added at different stages on traditional sparkling wines by solid-phase microextraction coupled to gas chromatography with mass spectrometric detection (SPME-GC-MS) [5]. Two times were considered, before and during tirage. The results showed that the addition of bentonite before this phase, in the base wine, had a lower effect on volatile compounds, with wines treated with 50% of the bentonite dosage in tirage with poor foam and aromatic characteristics. Also working on these type of wines (sparkling wines), and using solid-phase extraction-gas chromatography-mass spectrometry (SPE-GC-MS), Castro et al. established that the use of chitosan, a polysaccharide with fining, antimicrobial, antioxidant, and chelating properties, during the second fermentation, increased the protein (around 50%) and amino acid (9%) content of wines, without significant changes in polyphenols and organic acids. Esters increased as a consequence of this addition [6]. Amores-Arrocha, also thanks to the use of SPE-GC-MS, found that low bee pollen doses (0.1 and 0.25 g/L) before alcoholic fermentation could improve the aromatic profiles together with the odorant activity values levels in the production of red wines from Tintilla de Rota grape variety [7].

Ruiz et al., employing stir bar sorptive extraction coupled to gas chromatography and mass spectrometry (SBSE-GC-MS), studied the use of freezing techniques (ultrafast freezing, and liquid nitrogen freezing) in the wine-making of Muscat grapes [8]. The wines obtained using liquid nitrogen freezing exhibited higher levels of terpenoids, as well as higher levels of hydroxylic compounds and fatty acids than both the wines obtained through traditional methods and ultrafast freezing wines. In any case, both freezing techniques produced wines of a more intense aroma compared with those wines obtained by traditional methods.

The aging stage, both in wood and bottle, has also been considered in this special issue. Guerrero-Chanivet et al. carried out a study about the characterization of the aromatic profile of different wood chips (American oak, French oak, Spanish oak, Cherry and Chestnut) used for the aging of spirits and wines [9], whereas Vázquez-Pateiro et al. studied the evolution of volatile compounds, odor activity value-based aromatic notes, and sensory perception in Treixadura (*Vitis vinifera* L.) dry white wines during a 24-month bottle-aging period [10]. In this last study, most of the volatile compounds exhibited constant concentrations for 18 months in bottle, and after that significant and sharp decreases were observed.

In addition to these original research works, two reviews were also considered in this special issue. In one of them, Durán-Guerrero et al. have compilated all the different scientific works about the aroma of the Sherry oenological products (dry wines, sweet wines, vinegars, and brandies), with emphasis on the different analytical methodologies used [11]. In the other, the main secondary aroma compounds present in wine and the microorganisms involved in their presence were contemplated by Carpena et al. [12].

In summary, the Special Issue “*Novel Analysis on Aroma Compounds of Wine, Vinegar and Derived Products*” shows the great importance of sensory and analytical study of oenological products aroma and how they are influenced by the different stages and conditions under which they are elaborated.
